# Thin-Layer, Intermittent, Near-Infrared Drying of Two-Phase Olive Pomace: Mathematical Modeling and Effect on Recovery of Bioactive Compounds and Antioxidant Activity

**DOI:** 10.3390/foods14122042

**Published:** 2025-06-10

**Authors:** Ioanna Pyrka, Nikolaos Nenadis

**Affiliations:** 1 Laboratory of Food Chemistry and Technology, School of Chemistry, Aristotle University of Thessaloniki, 54124 Thessaloniki, Greece; ioannapyrka@chem.auth.gr; 2 Natural Products Research Centre of Excellence-AUTH (NatPro-AUTH), Center for Interdisciplinary Research and Innovation (CIRI-AUTH), 57001 Thessaloniki, Greece

**Keywords:** olive pomace, near-infrared drying, drying kinetics, mathematical modeling, antioxidants, bioactives, hydroxytyrosol

## Abstract

The present study examined the drying kinetics of two-phase olive pomace (OP) using near-infrared (NIR) thin layer intermittent drying at 70–140 °C. For the first time, this approach was combined with color, bioactive compound retention and antioxidant activity assessment. Among tested models, the Midilli’s semi-empirical model best described the drying behavior (*r*^2^ ≥ 0.99839, RMSE ≤ 0.01349). Effective diffusivity ranged from 1.417 × 10^−9^ to 5.807 × 10^−9^ m^2^/s, and activation energy was calculated at 23.732 kJ/mol. Drying at 140 °C reduced time by 68% compared to 70 °C. The corresponding sample had the highest total phenolics content, antioxidant activity (DPPH^●^, CUPRAC assays) and triterpenic acid (maslinic, oleanolic) content, and a significant amount of hydroxytyrosol, despite the increased sample browning. Compared to oven-drying (140 °C), NIR was equal or better and 3.2-fold faster. The same was evidenced compared to freeze-drying, except for tyrosol recovery (1.2-fold lower in NIR). These findings were obtained using two different OP industrial samples. Given that NIR is already used industrially for food drying, the present study offers proof-of-concept for its application as a rapid and eco-friendly pretreatment of OP for food and feed uses. However, scalability challenges and the limitations of semi-empirical modeling must be addressed in the future to support industrial-scale implementation.

## 1. Introduction

Olive pomace (OP) is a major by-product of the continuous extraction of olive oil from *Olea europaea* L. fruits. OP is produced in large quantities on a seasonal basis using different extraction technologies [1]. The OP obtained from two-phase decanter systems—gradually replacing the three-phase as eco-friendlier [1]—contains higher moisture (exceeding 65% *w*/*w*), due to the reduced water input and output associated with the two-phase process [2]. Consequently, the corresponding OP is more challenging to manage than the three-phase one. Traditionally, OP processing has mainly focused on residual oil recovery. However, it contains valuable bioactive compounds such as α-tocopherol, sterols, fatty acids, dietary fibers, phenolic compounds and triterpenic acids, representing a valuable source of functional compounds. Its extracts—or, more sustainably, its pulp-rich fractions—can be used to enhance oxidative stability, nutritional value and shelf-life of food products [3,4,5,6], while also contributing to the circular economy strategy aiming at achieving zero-waste.

Due to its high moisture content, OP is highly susceptible to microbial and enzymatic degradation. Therefore, immediate and appropriate drying is essential to extend its self-life and preserve its functional components for further utilization [7]. So far, various techniques have been employed, with hot air-drying being the most common [8,9,10,11,12,13,14,15,16,17]. Across studies, temperatures ranging from 40 to 150 °C have been employed. Most of the published papers have primarily focused on drying kinetics, neglecting the effects on bioactive constituents [8,9,10,13,14,17,18,19,20,21]. Others have only addressed bioactive compounds’ retention, mainly of polar phenols [2,11,15,16,22,23], and a few have attempted both [11,16]. Among them, there are efforts that explored innovative drying techniques such as microwave and infrared drying, which offer advantages in thermal efficiency. This is of high importance given the low thermal conductivity of OP during the falling-rate period of drying, which results in energy inefficiency and prolonged processing time when using conventional hot air- or gas-based methods [18,19,20,21,22].

Infrared (IR) drying is an efficient technique in which radiation is absorbed at the material’s surface, with near-infrared (NIR) wavelengths penetrating deeper into the sample [24,25]. Unlike conventional drying methods, IR heats the material directly and uniformly without reliance on ambient air, therefore eliminating the need for preheating [26]. Intermittent application further allows moisture and heat redistribution during tempering periods, reducing the overall drying and minimizing thermal degradation of sensitive bioactive compounds [27]. Additionally, IR drying has already been implemented at an industrial scale using tray or conveyor belt systems, making it a viable solution for large-scale biomass valorization. Previous studies on olive leaves [28,29] have demonstrated that high temperatures and short duration NIR drying can enhance the recovery of bioactive phenolic compounds, highlighting its potential for agrifood biomass valorization. Nevertheless, research on NIR drying of OP remains limited. To our knowledge, only two studies are relevant. Ruiz-Celma et al. [19] focused exclusively on modeling the drying behavior of a highly moist OP (~91%) exposed to temperatures between 80 and 140 °C (step of 20 °C), exploiting the precise control and the ability to record mass loss at short time intervals (0.5 min) of a NIR moisture analyzer. More recently, Castillo-Luna et al. [22] examined several drying techniques (freeze-, oven-, microwave- and infrared-drying) for their effects on phenolic content in experimental OP from two cultivars. However, the study did not clarify whether mid or near-infrared radiation was used, nor did it provide sufficient detail on sample thickness or the reason for the selected drying conditions (12 h, 60 °C).

Given these gaps, the present work aimed to investigate the drying of industrially sourced two-phase OP using intermittent NIR radiation applied to a thin sample layer with the aid of a moisture analyzer. The study focused on modeling the drying kinetics across a temperature range (70–140 °C), assessing the effect on color changes, and the levels of total and main compounds, namely the phenols hydroxytyrosol and tyrosol and the triterpenic acids (maslinic and oleanolic). The in vitro antioxidant potential of extracts from the dried OPs was further assessed employing DPPH^●^ and CUPRAC assays. The effectiveness of NIR drying was then compared with conventional oven- and freeze-drying techniques applied to two OP samples obtained from different cultivars and olive mills.

## 2. Materials and Methods

### 2.1. Chemicals and Standards

Chemicals and standards of appropriate purity (≥98%) were purchased. Hydroxytyrosol was from Extrasynthese (Lyon, France), tyrosol from Alfa Aezar (Kandel, Germany) and maslinic and oleanolic acids from Carbosynth Ltd. (Compton, UK). Gallic acid, quercetin, phosphoric acid, neocuproine, 6-hydroxy-2,5,7,8-tetramethyl-chroman-2-carboxylic acid (Trolox) and 1,1-diphenyl-2-picrylhydrazyl radical (DPPH^●^) were acquired from Sigma Chemical Co. (St. Louis and Burlington, MA, USA) and copper chloride was purchased from Merck KGaA (Darmstadt, Germany). HPLC grade methanol and water, ethanol (Disolol^®^), sodium carbonate and Folin–Ciocalteu reagent were sourced from Chem-Lab NV (Zedelgem, Belgium) and HPLC grade acetonitrile was obtained from Honeywell (Charlotte, NC, USA).

### 2.2. Materials

Olive pomace samples were collected from two different two-phase olive mills during the 2023 crop season. One sample (OP1) originated from Chalkidiki cv., while the second (OP2) from a mixture of Chalkidiki, Megaritiki and Kalamon cv. OP1 was provided by a mill located in Chalkidiki prefecture and OP2 from a similar mill in the Kavala prefecture during mid-October and mid-November, respectively. Immediately after collection, the samples were transferred into 3 L containers without headspace and frozen at −20 °C until further use. The initial mean moisture content was 69% and 70% on a dry basis (dw), as determined using a DAB 100-3 NIR moisture analyzer (Kern & Sohn, GmbH, Balingen, Germany) at 105 °C until constant weight was achieved.

### 2.3. Drying Assays

The drying kinetics study was conducted on OP1 using the same NIR moisture analyzer, at temperatures of 70, 90, 100, 120 and 140 °C. This wide temperature range was selected to observe meaningful variations in diffusivity with temperature and to ensure a robust linear fit in the Arrhenius plot (vide infra). The stepwise increase of 10 or 20 °C balances experimental feasibility and kinetic sensitivity. The intermediate temperature of 100 °C was included on purpose considering the work of Derardja et al. [30], where the thermal stability behavior of isolated olive phenoloxidase was examined. Moreover, the selected range closely aligns with that used in a comparable drying kinetics study (80–140 °C) [19], reinforcing the relevance of our temperature selection. The inclusion of 70 and 140 °C was further supported by preliminary findings from a previous study realized by our group [31]. Approximately 15 g of sample was uniformly spread in the pan analyzer to a thickness of 0.5 cm to cover the entire pan till the edges. The latter falls within the range of thickness values commonly used in thin-layer OP drying studies [10,31]. The moisture loss (g) was recorded every 2 s (total recorded points: 4102 for 70, 3132 for 90, 2307 for 100, 1460 for 120 and 1303 for 140 °C), using the software Balance Connection software SCD-4.0 (version 4.2.4) provided by the manufacturer. Both samples (OP1, OP2) were used to assess the NIR drying efficiency at 140 °C compared to the most widely used techniques, oven-drying (Heraeus, Hana, Germany; 140 °C; 140 min) and freeze-drying (ALPHA 1–2 LDplus, MartinChrist, Germany; −50 °C; 0.1 mbar; 24 h). The dried OP samples were then milled with a blender and sieved to obtain pulp-rich fractions smaller than 500 μm in diameter. Each process was repeated in triplicate.

### 2.4. Drying Kinetics Study

The dimensionless moisture ratio (*MR*) was calculated to study the NIR drying process, using the following equation:

(1) $$MR=\frac{m_{t}-m_{e}}{m_{0}-m_{e}}$$

where *m_t_* is the mass (g) of sample at time *t*, *m*_0_ is the initial sample mass and *m_e_* is the mass in equilibrium.

The experimental values of MR were fitted to the three semi-empirical models that best described the procedure, as shown by Ruiz-Celma et al. [19], namely the Page [32], Logarithmic [33] and Midilli [34] models. Non-linear regression techniques were used to obtain the constraints of the models, using Origin Pro 7.5 software (Northampton, MA, USA) based on the Lavenberg–Marquardt method. The fitting of the experimental to the theoretical values was evaluated using coefficient of determination (*r*^2^), reduced chi-square (χ^2^) and root mean square error (RMSE).

As the drying time is controlled by the internal resistance to mass transfer, due to the presence of the falling rate period, the experimental results can be interpreted by Fick’s diffusion equation:

(2) $$\frac{dMR}{dt}=D_{eff}\times \frac{d^{2}M}{dr^{2}}$$

Neglecting the internal moisture movement within the thin layer, moisture transfer can be treated as a one-dimensional diffusion process occurring from the bottom to the upper surface of the sample. The diffusion analysis is based on the following assumptions: (i) the initial moisture content of the sample is uniformly distributed, (ii) moisture migration follows a diffusion pattern, (iii) shrinkage is insignificant and (iv) the diffusion coefficient remains constant at each temperature.

The following equation of Crank [35] can be used for samples with planar geometry:

(3) $$MR=\frac{8}{\pi^{2}}\sum_{n=0}^{\infty }\frac{1}{{\left(2n+1\right)}^{2}}exp\left(-\frac{{\left(2n+1\right)}^{2}\pi^{2}D_{eff}t}{4L^{2}}\right)$$

where *D_eff_* is the diffusion coefficient (m^2^/s) and *L* the sample thickness (m).

The linear solution of the equation is obtained using a simple approximation, assuming that for extended drying periods (MR < 0.6), only the first term of the series equation is significant [36]. After taking the natural logarithm in both members of the equation, the result is the following:

(4) $$lnMR=ln\frac{8}{\pi^{2}}-\frac{\pi^{2}\;D_{eff}\;t}{4L^{2}}$$

*D_eff_* was calculated, from the slope of the graph *lnMR*, using the experimental MR values for each temperature, versus time of drying:

(5) $$Slope=\frac{\pi^{2}\times D_{eff}}{4\;L^{2}}$$

The relationship between drying conditions and the determined values of effective diffusivity can be expressed using an Arrhenius-type equation:

(6) $$D_{eff}=D_{0}\times exp\frac{-E_{a}}{RT}$$

where *D*_0_ is the pre-exponential factor (m^2^/s), *E_a_* the activation energy (kJ/mol), *R* the universal gas constant (kJ/mol K) and *T* the absolute temperature (K). Using the logarithmic form of the latter equation and plotting ln*D_eff_* against 1/T, *D*_0_ and *E_a_* can be calculated from the *y*-axis intercept and the slope of the line, respectively.

### 2.5. Color Evaluation

CIE L*a*b* color measurements were carried out in the pulp-fraction of dried OP samples, using a Hunter Miniscan XE Plus spectrophotometer (Hunter Laboratories, Reston, VA, USA). The rectangular coordinates L*, a* and b*, corresponding to darkness (0)/lightness (100), greenness (−)/redness (+) and blueness (−)/yellowness (+), respectively, were automatically calculated by the spectrophotometer after proper calibration against standard white and black plates. Five replicate measurements were taken for each sample and the average values were converted to the closest standard color using open-access online software available at https://www.e-paint.co.uk/convert-lab.asp (accessed on 13 February 2025), given that the available instrument did not provide any visualization capability.

### 2.6. Determination of Bioactive Compounds and Antioxidant Activity

Total phenolics content (TPC), antioxidant activity (AA) and selected phenolic and triterpene compounds were determined in the extracts of dried olive pomace. To obtain the extracts, a portion (1 g) of dried OP pulp-rich fraction was mixed with 20 mL of methanol and macerated in an ultrasound bath (P 30H, Elmasonic, Elma) for 30 min, at a temperature below 30 °C. Centrifugation was then applied (10,000× *g* for 10 min) and the collected supernatant was stored at −20 °C till analysis [31].

#### 2.6.1. Total Phenolics Content (TPC)

Folin–Ciocalteu assay was used to determine the total phenolics content of dried OPs. In brief, an aliquot (100 μL) of each extract was mixed with 1.5 mL of Na_2_CO_3_ (20% *w*/*v*) and after 3 min, 8.4 mL of deionized water was added. The mixture was kept in the dark for 1 h and measurements were recorded against a blank at 750 nm. The results were expressed as gallic acid equivalents (mg GAE/kg dw) [31].

#### 2.6.2. Antioxidant Activity (AA)

Antioxidant activity of dried OPs was estimated using DPPH^●^ and CUPRAC assays, according to [37,38]. For DPPH^●^, an aliquot (80 μL) of each extract was mixed with 2.8 mL of a 0.1 mM DPPH^●^ methanolic solution and incubated in the dark for 30 min. Absorbance measurements were recorded at t = 0 and at the endpoint at 516 nm to calculate the % radical scavenging activity. In the case of CUPRAC assay, an aliquot (100 μL) of each extract was mixed with 1 mL each of Cu(II), neocuproine and ammonium acetate buffer (pH 7) solutions. The volume was adjusted to 4.1 mL with deionized water. After 1 h of reaction, absorbance was recorded at 450 nm. Appropriate blanks were used for correction in each assay and results were expressed as Trolox equivalents (mol TE/kg dw).

#### 2.6.3. Determination of Phenolics and Triterpenic Acids

Phenolic compounds (hydroxytyrosol and tyrosol) and triterpenic acids (maslinic and oleanolic acids) were quantified at 280 and 210 nm, respectively, using a UHPLC system (Nexera X2, Shimadzu, Kyoto, Japan) with a SIL-30 AC autosampler, LC-30b CE pump, CTO-20 AC oven, SPD-M20A diode array, RF-20AXS fluorescence detector and an Accucore C18 column (100 mm × 4.6 mm × 2.6 µm, Thermo Scientific, San Jose, CA, USA).

In brief, hydroxytyrosol (HT) and tyrosol (T) were determined in a gradient elution mode using as mobile phase (A) acidified water (0.2% H_3_PO_4_) and (B) acetonitrile: 0 min, 10% B; 4 min, 20% B; 17.2 min, 50% B; 19.2, 95% B; 20.8 min, 95% B; 24 min, 10% B. The flow rate was set at 0.5 mL/min and injection volume at 5 μL [6]. Oleanolic (OA) and maslinic (MA) acids were quantified in isocratic mode using as mobile phase a mixture of acidified water (0.2% H_3_PO_4_) and methanol (92:8 *v*/*v*), a flow rate of 0.8 mL/min and an injection volume of 5 µL [6]. The identification was based on matching DAD spectra and peak retention time using the corresponding analytical standards. All results were given as g/kg dw with the aid of external calibration curves. The LOD (3 S/N)), and LOQ (10 S/N) values for the target analytes were: 0.15/0.45 mg/µL, HT; 0.06/0.19 mg/µL, T; 2.0/6.0 mg/µL for both OA and MA.

### 2.7. Statistical Analysis

Each measurement was carried out in triplicate for each sample (color was measured five times) and the results are presented as mean values ± standard deviation. SPSS Statistics v.28.0 software (IBM Corporation, Armonk, NY, USA) was used to examine the homogeneity of variances using Levene’s test, applying Welch ANOVA (which does not assume equal variances and is more robust under conditions of heteroscedasticity or non-normality) and estimating the effect size through Eta-squared calculation and Games–Howell post hoc test at *p* < 0.05.

## 3. Results and Discussion

### 3.1. Drying Kinetics Analysis

**Figure 1:**
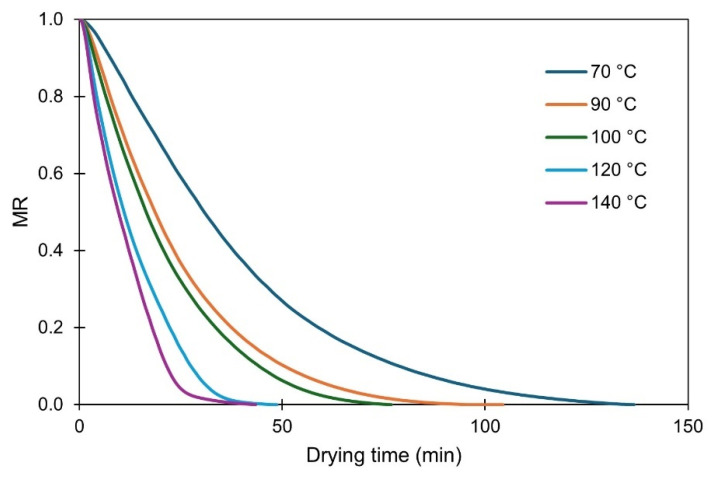
Experimental drying curves of olive pomace at five studied temperatures. Plots represent average of three measurements.

The drying curves (including the total recorded points) of OP1 at 70, 90, 100, 120 and 140 °C are shown in Figure 1. As the drying temperature increases, the slope of the exponential curve becomes steeper, indicating a reduction in the overall drying time. Similar behavior was reported by Ruiz-Celma et al. [19] for NIR dried OP, although the data recording was terminated at a residual moisture content of 8.69% without reaching mass equilibrium (MR = 0).

**Figure 2:**
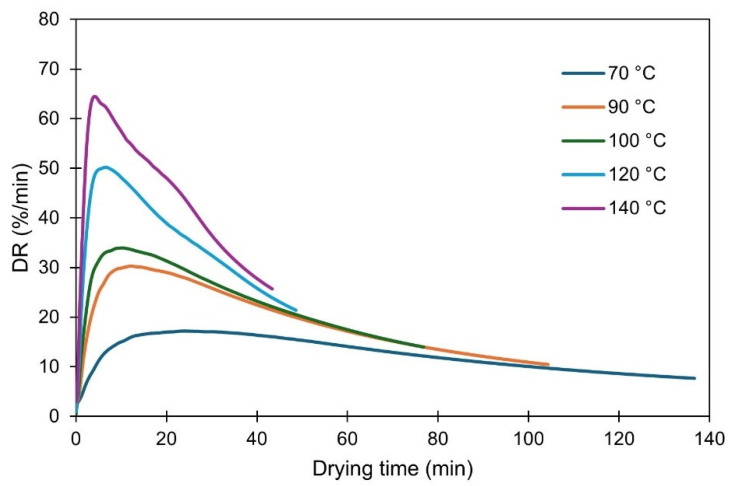
Experimental drying speed curves of olive pomace at five studied temperatures. Plots represent average of three measurements.

The drying rate (DR, %/min) curves at the five temperatures are shown in Figure 2. Upon the initial phase of drying, positive slopes indicate an acceleration in the drying rate, coinciding with the sample’s temperature increase and enhanced water evaporation from the surface. This initial phase was shorter at higher temperatures. As surface moisture was reduced, a deceleration phase followed, due to the slower diffusion rate of internally located water to the surface. At higher temperatures, the drying rates increased as evidenced by the higher peaks of the curves, reducing the time to reach equilibrium. The equilibrium was attained at 43 min for 140 °C, while it took approximately 136 min at 70 °C (2.5-fold increase). No constant DR period was observed at any temperature, probably due to uneven surface moisture distribution, as previously explained by Gomez-Delgado et al. [39]. Our results are in line with those of Ruiz-Celma et al. [19].

### 3.2. Modeling of Drying Curves

Ruiz-Celma et al. [19] evaluated 13 drying models for NIR drying of OP from a two-phase olive mill and concluded that Midilli’s model provided the best fit, with an *r*^2^ value of 0.9999 for temperatures between 80–140 °C. Thus, in this study, the same model was tested for the OP1 sample, alongside the Page and logarithmic models. The latter two have also demonstrated higher predictive accuracy for drying OP in a cabinet dryer at 50–110 °C [8,9]. The model equations, calculated constants from the experimental data and curve-fitting criteria are summarized in Table 1. All three models presented high *r*^2^ values (>0.996), indicating a good fit. However, Midilli’s model exhibited the highest *r*^2^ values (≥0.99839) and the lowest RMSE (≤0.01349), and thus was considered the most suitable. The fitted curves are shown in Figure S1.

### 3.3. Effective Diffusivity and Activation Energy

**Figure 3:**
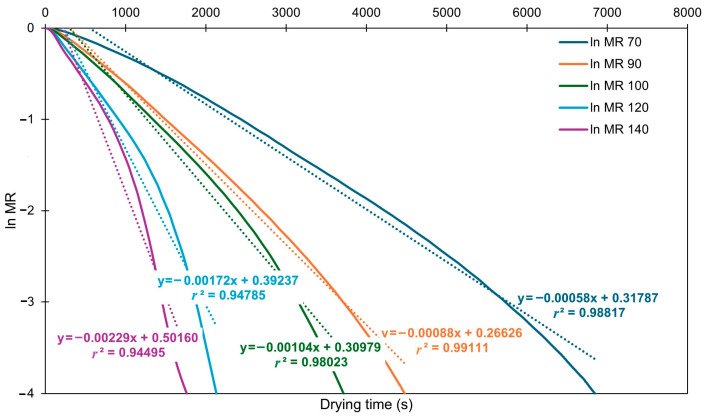
Plot of ln(MR) against drying time at studied temperatures (solid lines). Linear trendlines (dotted lines), their equation and R^2^ values are displayed for each curve. Plots represent average of three measurements.

The effective diffusivity (*D_eff_*) values calculated from the slopes (Figure 3) are shown in Table S1. Values ranged from 1.417 × 10^−9^ to 5.807 × 10^−9^ m^2^/s and increased with temperature. These results were slightly lower than those reported by Ruiz-Celma et al. [19], who found values in the range of 6–16 × 10^−9^ m^2^/s for NIR drying at 80–140 °C. The observed discrepancy may stem from differences in initial moisture content, as their samples had significantly higher initial moisture content (91.9%).

**Figure 4:**
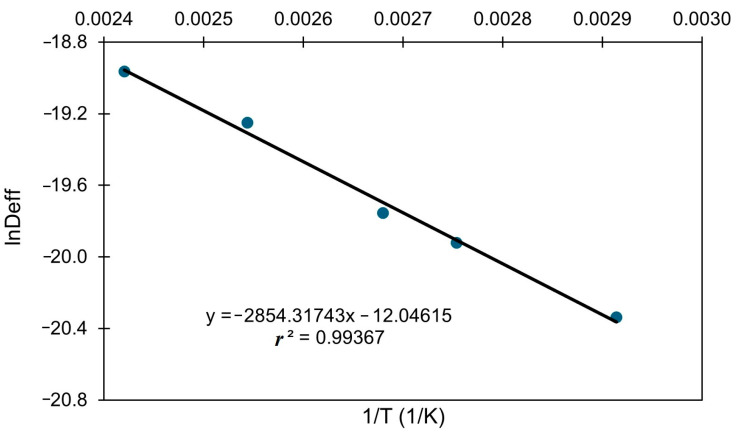
Arrhenius-type relationship between effective diffusivity (*D_eff_*) and temperature (T, K).

The plotting of ln*D_eff_* values versus T^−1^ (K^−1^) (Figure 4) enabled the calculation of activation energy (*E_a_*) for moisture diffusion, yielding a value of 23.732 kJ/mol. This value was close to that (21.30 kJ/mol) reported by Ruiz-Celma et al. [19], who used only four temperatures and obtained an *r*^2^ value of 0.959. The pre-exponential coefficient (*D*_0_) was calculated to be 5.9 × 10^−6^ m^2^/s, compared to 8.285 × 10^−6^ m^2^/s in the earlier study of Ruiz-Celma et al. [19].

### 3.4. Temperature Effect on Color and Chemical Composition

The color measurement was conducted on pulp-rich fractions obtained from OP1 after removing the pit fragments for uniformity purposes. Based on our previous experience with fresh olive leaves [29], where the chemical characterization did not accurately reflect the material’s true potential—and considering technical limitations related to homogenization and sieving—the results presented and discussed here refer exclusively to dried samples. The corresponding values for these dried samples are shown in Table 2. Levene’s test results (df1 = 4, df2 = 10, *p* = 0.108–0.324) indicated that the assumption of homogeneity of variances was met for the three variables. The Welch’s ANOVA—a robust test that does not assume equal variances—revealed statistically significant differences *(p* = 0.000, df1 = 4, df2 = 4.135–4.766) in group means across all three variables, suggesting that group membership significantly impacts the findings. The latter was verified by effect size which was very high (Partial Eta squared = 0.998–0.999). Regarding the findings after the application of Games–Howell post hoc test, it was evidenced that dried samples at 70–120 °C presented similar luminosity values (Table 2) although statistical differences were observed. Both a^*^ and b^*^ values increased slightly with temperature, indicating a shift toward red and yellow hues, respectively and samples exhibited a light brown appearance as evidenced by the visualization approach adopted. At 140 °C, L* and b* values decreased significantly, producing a darker, less yellow product.

The same statistical evaluation was applied to the values obtained for the total phenolics content, antioxidant activity and the key constituents measured with HPLC. Homogeneity was verified in all variables (df1 = 4, df2 = 10, *p* = 0.072–0.854). The Welch’s ANOVA indicated that for TPC and the two different antioxidant activity assays (DPPH^●^, CUPRAC) there were no statistical differences (*p* = 0.19, 0.112, 0.258) but the effect size (Partial Eta squared = 0.690, 0.601, 0.466) suggested moderate to large effects. Regarding the two phenols and the triterpenic acids, homogeneity was verified (df1 = 4, df2 = 10, *p* = 0.189–0.495), as well as statistical difference (*p* = 0.000), whereas very large effect sizes were obtained (Partial Eta squared = 0.989–0.998).

**Figure 5:**
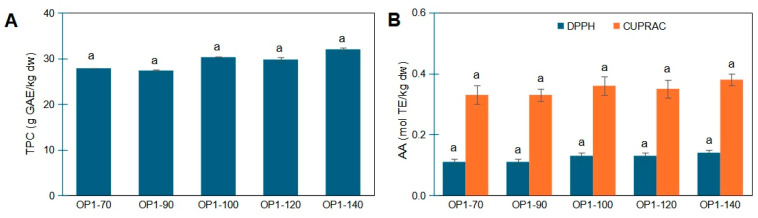
(**A**) Total phenolics content (TPC) and (**B**) antioxidant activity (AA), using DPPH^●^ and CUPRAC assays, of olive pomace (OP1) dried at five different temperatures (70, 90, 100, 120, 140 °C). Results are expressed as mean ± standard deviation values (*n* = 3). Same letters above bars indicate that no significant difference was found at (*p* < 0.05) applying Games–Howell post hoc test.

Thus, applying the Games–Howell post hoc test, no statistical difference was evidenced across means, for TPC and AA evaluation (Figure 5). Considering the effect size, an increasing trend was observed in TPC values (Figure 5A), reaching a maximum (31.99 g GAE/kg dw) at 140 °C. Such finding is in accordance with the results of Ahmad-Qasem et al. [40] who reported that temperature had no significant influence on TPC under forced air drying, even though they observed a minor increasing tendency at temperatures higher than 70 °C, linking this to the possible thermally induced phenolic formation from precursor molecules. In our previous study [31], analysis of samples dried at 70 and 140 °C in the premises of an agricultural cooperative showed that those dried at the higher temperature had an almost 2-fold higher TPC content. Other studies have shown mixed results [2,11,12,15], with some indicating no significant differences between 75 °C and 100 °C, but generally higher values than drying at 50 °C. Similarly, antioxidant activity using both DPPH^●^ and CUPRAC assays (Figure 5B) showed an increasing trend reaching maximum levels (0.14 and 0.38 mol TE/kg dw, respectively) at 140 °C. Ahmad-Qasem et al. [40], who studied oven-dried OP at 50–150 °C, found a significant AA increase (15.5%) only at 150 °C using the FRAP assay.

**Figure 6:**
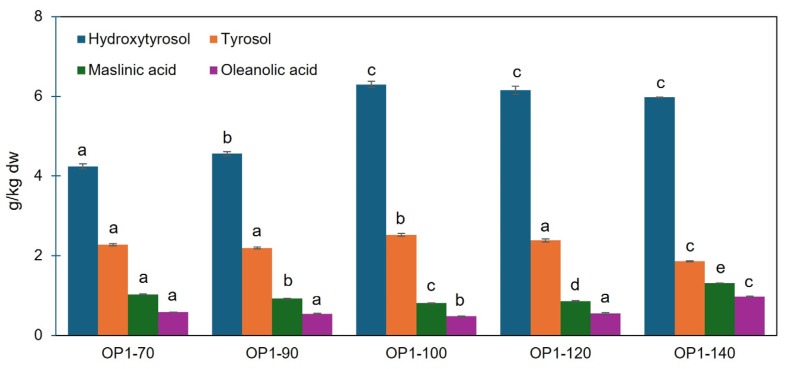
Main phenolic compounds (hydroxytyrosol and tyrosol) and triterpenic acids (maslinic and oleanolic acids) of olive pomace (OP1) dried at five different temperatures (70, 90, 100, 120, 140 °C). Results are expressed as mean ± standard deviation values (*n* = 3). Different letters above bars indicate a significant difference (*p* < 0.05) applying Games–Howell post hoc test.

HPLC analysis (Figure 6) revealed that hydroxytyrosol and tyrosol levels were the highest when OP1 was dried at 100 °C (6.29 and 2.52 g/kg dw respectively). From 70 to 100 °C both compounds increased, particularly hydroxytyrosol, which was at 100 °C 1.38-fold and 1.48-fold higher than at 90 °C and 70 °C, respectively. The moderate decrease observed at higher temperatures for hydroxytyrosol was not significant. Tyrosol, decreased in the process involving drying at 140 °C compared to 70 °C despite the significantly lower treatment time. Although such a finding is rather surprising considering that tyrosol is a monophenol, it can be inferred from the findings that it should degrade at 140 °C. The pattern for hydroxytyrosol aligns with the findings by Loschi et al. [2] for samples dried at 50, 75 and 100 °C, as the levels at the higher temperature were 1.3-fold and 4.4-fold higher than those at 75 °C and 50 °C, respectively. Present findings are partially in agreement with those of our past study [31], where oven drying at 70 °C was detrimental to the levels of the two phenols. Negative impact was observed especially on the more abundant hydroxytyrosol, when compared to the dried samples at 140 °C, as in the latter, hydroxytyrosol was almost 10-fold higher, and tyrosol 2-fold. The reduced phenol content was likely due to ineffective enzyme deactivation, in agreement with the observations of Derardja et al. [30] for the olive phenoloxidase activity at 70 °C, and to insufficient cell-tissue disruption, which is a prerequisite for the release of target compounds.

In triterpenic acids, small but statistically significant differences were observed across 70–120 °C for maslinic acid, whereas for oleanolic acid, the values were almost comparable. At 140 °C, the highest levels were observed for both acids (1.31 and 0.97 g/kg dw for maslinic and oleanolic acid, respectively). These findings support the notion that triterpenic acids are more thermally stable than phenolics and less susceptible to enzymatic oxidation [29]. The reason for the lower levels found at 70 °C compared to 140 °C remains unclear. Other studies [2,31] have also shown inconsistent correlations between drying temperature and triterpene levels. Nonetheless, based on the present findings, it can be hypothesized that at 140 °C the increased disruption of sample tissues—particularly the skin tissues, where triterpenic acids are primarily located—enhanced the extraction efficiency.

### 3.5. Drying Technique Effect on Color and Chemical Composition

Drying OP1 at 140 °C using IR resulted in the shortest processing time (approximately 3.25 times less than OD), reflecting efficient heat transfer. Homogeneity was verified in all variables (df1 = 2, df2 = 6, *p* = 0.059–1.000) for OP1. The Welch’s ANOVA indicated that for all the variables statistical differences were found (*p* = 0.000–0.010) and very large effect sizes (Partial Eta squared = 0.861–0.998) were observed. Regarding OP2, which was used to obtain preliminary insights into possible cultivar-dependent drying responses, the homogeneity was acceptable in all variables (df1 = 2, df2 = 6, *p* = 0.052–1.000) and statistical treatments were found except for TPC and AA (*p* = 0.096, 0.191, 0.089). Consequently, for the latter, moderate effect sizes in terms of Partial Eta squared values were found (0.628, 0.500, 0.589), whereas for color (0.94–1.00) and key bioactives (0.961–0.999) were very large.

Color in OD samples appeared similar to that of IR-dried ones (Table 3) despite the statistical differences in all three variables. FD samples exhibited greater luminosity and yellowness but lower redness, resulting in a beige appearance. Sinrod et al. [16] reported similar findings, albeit with smaller differences, possibly due to lower OD temperature (80 °C). Cecchi et al. [23] noted that browning was consistent across OD temperatures (50–110 °C), possibly due to chemical or enzymatic reactions occurring at temperatures above 50 °C, a hypothesis which—at least regarding olive phenoloxidase—is in line with the findings of Derardja et al. [30]. Nevertheless, when the authors used an industrial drier instead of a laboratory oven and employed 150 °C, the sample obtained did not differ in color to the FD, due to the fast procedure.

**Figure 7:**
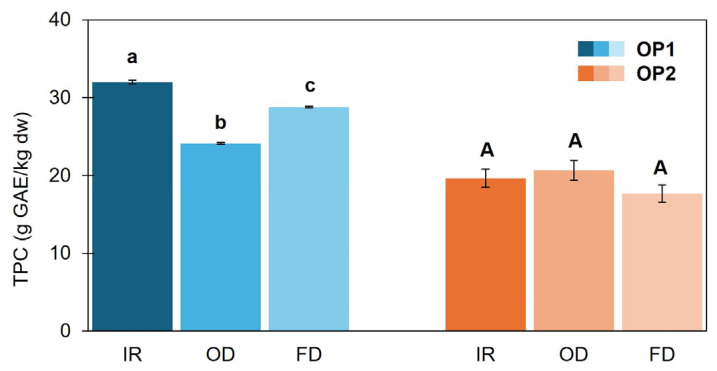
Total phenolics content (TPC) of olive pomace samples (OP1, OP2) dried with infrared radiation at 140 °C (IR), oven heating at 140 °C (OD) and freeze-drier (FD). Results are expressed as mean ± standard deviation values (*n* = 3). Different lowercase and capital letters above bars indicate a significant difference (*p* < 0.05) for OP1 and OP2, respectively, applying Games–Howell post hoc test.

TPC levels (Figure 7) were higher in OP1-IR (31.99 g/kg dw), followed by OP1-FD (26.16 g/kg dw) and OP1-OD (24.11 g/kg dw), whereas no differences were obtained for OP2.

**Figure 8:**
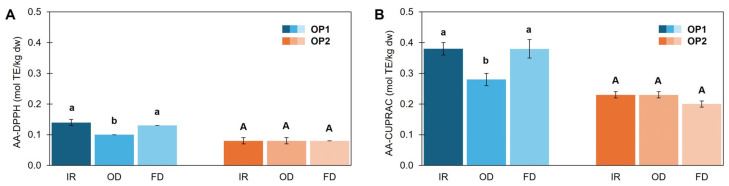
Antioxidant activity (AA) of olive pomace samples (OP1, OP2), measured by DPPH● (**A**) and CUPRAC (**B**) assays and dried with infrared radiation at 140 °C (IR), oven heating at 140 °C (OD) and freeze-drier (FD). Results are expressed as mean ± standard deviation values (*n* = 3). Different lowercase and capital letters above bars indicate a significant difference (*p* < 0.05 for OP1 and OP2, respectively, applying Games–Howell post hoc test.

The AA using both assays (Figure 8) was almost similar in OP1-IR and OP1-FD, and approximately 1.4-fold higher than that of the OP1-OD sample, whereas in OP2 no differences were observed among treated samples. These results partially agree with literature, as Sinrod et al. [16] found slightly better DPPH^●^ scavenging for the OD sample at 80 °C compared to FD. In the work of Difonzo et al. [4], both TPP and AA were much higher (almost 2-fold) in the FD sample compared to an OD sample dried at 120 °C.

**Figure 9:**
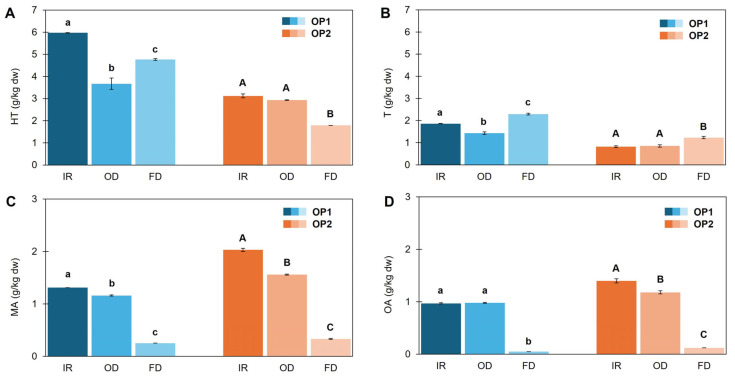
Main phenolic compounds (hydroxytyrosol, **A** and tyrosol, **B**) and triterpenic acids (maslinic, **C** and oleanolic, **D** acids) of olive pomace samples (OP1, OP2) dried with infrared radiation at 140 °C (IR), oven heating at 140 °C (OD) and freeze-drier (FD). Results are expressed as mean ± standard deviation values (*n* = 3). Different lowercase and capital letters above bars indicate a significant difference (*p* < 0.05) for OP1 and OP2, respectively, applying Games–Howell post hoc test.

HPLC analysis (Figure 9) revealed that hydroxytyrosol was highest in OP1-IR (5.97 g/kg dw), while tyrosol was more abundant in OP1-FD (2.29 g/kg dw). OP2-IR was equal to OP2-OD concerning the levels of the two phenols. FD was more effective only concerning tyrosol, findings rather surprising considering its lower tendency to oxidize compared to hydroxytyrosol [37] and its higher thermal stability [41]. Even so, considering also the findings from Section 3.4, a thermal instability of tyrosol can be inferred. Triterpenic acids were found at higher levels in IR and OD samples, whereas in FD up to 81% and 95% lower levels of maslinic and oleanolic acids were evidenced, respectively. These results are unexpected given the generally recognized thermal stability of triterpenic acids, although substrate structure may play a role [29]. Given that FD is a mild dehydration technique and in light of the findings in Section 3.4, it can be deduced that sample tissues—particularly the skin, which is rich in triterpenic acids—underwent less disruption. As a result, the extraction efficiency of these compounds from the FD sample was reduced.

Literature comparisons are variable. Sinrod et al. [16] found higher hydroxytyrosol and tyrosol levels in the OD (by 2.8-fold and 1.5-fold respectively) than the FD sample. Cecchi et al. [23] reported for the two phenols that the highest levels were at 110 °C upon drying experiments in the range of 50 to 110 °C, though still lower (by 1.4- and 1.3-fold respectively) than the FD sample. Castillo-Luna et al. [22] showed that IR was superior for preserving certain phenol glycosides (e.g., hydroxytyrosol glucoside), while OD was optimal for hydroxytyrosol. FD performed better for the retention of oleacein and oleuropein aglycone. The same authors, upon measuring the triterpenic acids in the olive fruit instead of the pomace, realized that both maslinic and oleanolic acids were found at higher levels with IR drying. FD was less efficient, possibly attributed “to the lower compatibility of the sample type to oven and freeze-drying equipment” according to the authors without further clarification. Considering that all these examinations are tedious, future studies are needed to integrate into the IR drying process, non-invasive spectroscopic techniques and chemometrics for real-time monitoring of the effect on bioactives [42] to expedite decision-making on drying conditions.

## 4. Conclusions

The post-harvest treatment of OP, namely the drying process, is a critical aspect, as it affects the content of bioactive compounds such as polar phenols and triterpenic acids, components of high importance for value-added applications. Furthermore, the economic viability of the treatment is essential, particularly in providing fast and efficient manipulation of the substantial quantities produced seasonally by the olive mills. The present work serves as a proof-of-concept for the use of intermittent NIR thin-layer drying to treat OP that derives from a two-phase decanter system, demonstrating for first time its capability to provide a material with significant levels of bioactives (polar phenols and triterpenic acids) for first time.

The NIR drying outperformed conventional oven drying by reducing the processing time by 3.2-fold at 140 °C and providing powders with significant amount of bioactives, although further optimization is required to maximize the obtained levels across all target compounds. These findings align with published reports advocating high temperature and short-duration drying.

In addition to demonstrating technical feasibility, the study also addressed the modeling of drying kinetics which was efficiently described by the Midilli’s model. While the model provided a good fit for the experimental data and may be useful for preliminary plant design, as a semi-empirical model, it presents inherent limitations in fully describing the complex heat and mass transfer phenomena under variable scaling conditions. Future work should validate and refine the model under pilot and industrial-scale setups. Furthermore, industrial-scale equipment should address engineering and economic challenges, such as the achievement of uniform irradiation and efficient energy use across large batches coupled to the effect of intermittent irradiation on product quality and drying efficiency. Nonetheless, the reduced drying time is a potential cost advantage over conventional oven-drying due to lower energy consumption and faster throughput. Thus, despite the higher upfront expenses, the cost–benefit ratio should favor NIR. A comprehensive cost–benefit analysis under scaled-up conditions will be essential to evaluate the full economic impact of implementing intermittent NIR drying technology in olive mill by-product valorization strategies.

## Figures and Tables

**Table 1:** Constants of studied drying models and curve fitting criteria for olive pomace calculated by experimental data.

Model	Analytical Expression	Temperature	Model Constants	*r* ^2^	χ^2^	RMSE
Page	MR = exp(−kt^y^)	70 °C	k = 0.00004, y = 1.30859	0.99978	0.00002	0.02051
		90 °C	k = 0.00012, y = 1.23584	0.99964	0.00003	0.12801
		100 °C	k = 0.00014, y = 1.23332	0.99928	0.00006	0.17871
		120 °C	k = 0.00016, y = 1.28743	0.99670	0.00029	0.34259
		140 °C	k = 0.00017, y = 1.28435	0.99623	0.00034	0.35095
Logarithmic	MR = a exp(−kt) + c	70 °C	a = 1.14427, k = 0.00041, c = −0.04889	0.99688	0.00027	0.01690
		90 °C	a = 1.11630, k = 0.00069, c = −0.03087	0.99839	0.00013	0.15309
		100 °C	a = 1.12468, k = 0.00072, c = −0.05769	0.99894	0.00009	0.20357
		120 °C	a = 1.15699, k = 0.00104, c = −0.08896	0.99729	0.00024	0.35777
		140 °C	a = 1.18179, k = 0.00098, c = −0.12324	0.99845	0.00014	0.36377
Midilli	MR = a exp(−kt^n^) + bt	70 °C	a = 1.01384, k = 0.00005, *n* = 1.26512, b = −8.9095 × 10^−7^	0.99992	0.00001	0.00943
		90 °C	a = 1.02388, k = 0.00018, *n* = 1.17911, b = −1.3168 × 10^−6^	0.99990	0.00001	0.00348
		100 °C	a = 1.01857, k = 0.00024, *n* = 1.15943, b = −4.9527 × 10^−6^	0.99988	0.00001	0.00560
		120 °C	a = 1.01421, k = 0.00031, *n* = 1.18674, b = −0.00001	0.99839	0.00014	0.01349
		140 °C	a = 1.01980, k = 0.00039, *n* = 1.14839, b = −0.00002	0.99887	0.00010	0.01238

**Table 2:** Color coordinates (L*, a*, b*) of pulp-rich fraction of olive pomace (OP1) dried at five different temperatures (70, 90, 100, 120, 140 °C), followed by their color visualization.

Sample	L*	a*	b*	Color Visualization *
OP1-70	32.28 ± 0.13 a	10.50 ± 0.17 a	21.54 ± 0.55 a	
OP1-90	30.09 ± 0.02 b	12.11 ± 0.05 b	21.54 ± 0.14 a	
OP1-100	32.70 ± 0.17 c	13.64 ± 0.14 c	24.98 ± 0.30 b	
OP1-120	30.53 ± 0.18 d	16.21 ± 0.06 d	25.77 ± 0.37 c	
OP1-140	18.80 ± 0.27 e	11.82 ± 0.10 e	13.13 ± 0.48 d	

Results are expressed as mean ± standard deviation values (*n* = 5). Different letters indicate a significant difference (*p* < 0.05) applying Games–Howell post hoc test. * Color visualization was via conversion of experimental color coordinates to nearest color with an open-access software (see 2.5).

**Table 3:** Color coordinates (L*, a*, b*) of pulp-rich fraction of olive pomace (OP1, OP2) dried with infrared radiation at 140 °C (IR), oven heating at 140 °C (OD) and freeze-dried (FD), followed by their color visualization.

Sample	L*	a*	b*	Color Visualization *
OP1-IR	18.80 ± 0.27 a	11.82 ± 0.10 a	13.13 ± 0.48 a	
OP1-OD	19.55 ± 0.29 a	12.19 ± 0.37 b	17.01 ± 0.11 b	
OP1-FD	47.63 ± 0.39 b	5.58 ± 0.16 c	26.69 ± 0.55 c	
OP2-IR	13.27 ± 0.16 A	5.69 ± 0.63 A	9.09 ± 1.45 A	
OP2-OD	16.35 ± 0.22 B	10.51 ± 0.81 B	12.81 ± 0.57 A	
OP2-FD	41.08 ± 0.19 C	8.64 ± 0.28 B	23.16 ± 0.55 B	

Results are expressed as mean ± standard deviation values (*n* = 5). Different lowercase and uppercase letters indicate a significant difference (*p* < 0.05) in OP1 and OP2 samples, respectively, applying Games–Howell post hoc test. * Color visualization was via conversion of experimental color coordinates to nearest color with an open-access software (see 2.5).

## Data Availability

The original contributions presented in the study are included in the article/Supplementary Material; further inquiries can be directed to the corresponding author.

## Supplementary Materials

The following supporting information can be downloaded at: https://www.mdpi.com/article/10.3390/foods14122042/s1, Figure S1: Page (A), logarithmic (B) and Midilli (C) kinetic models adjusted to experimental drying curves at 70, 90, 100, 120 and 140 °C; Table S1: Effective diffusivity at studied temperatures.

## Abbreviations

The following abbreviations are used in this manuscript:

AA	antioxidant activity
DR	drying rate
FD	freeze-dried
H	hydroxytyrosol
IR	infrared-dried
MA	maslinic acid
OA	oleanolic acid
OD	oven-dried
OP	olive pomace
T	tyrosol
TPC	total phenolics content
