# Supplementary material for: Thin-Layer, Intermittent, Near-Infrared Drying of Two-Phase Olive Pomace: Mathematical Modeling and Effect on Recovery of Bioactive Compounds and Antioxidant Activity
Source: Foods. 2025 Jun 10;14(12):2042. doi: 10.3390/foods14122042 (PMC12191536; doi:10.3390/foods14122042)
Supplement: Supplementary file 1 [file foods-14-02042-s001.zip › foods-3632751-supplementary.pdf]

SUPPLEMENTARY MATERIAL

Thin-layer intermittent near-infrared drying of two-phase olive pomace: Mathematical modeling and effect on recovery of bioactive compounds and antioxidant activity

Ioanna Pyrka[1], Nikolaos Nenadis[1,2]*

[1]Laboratory of Food Chemistry and Technology, School of Chemistry, Aristotle University of Thessaloniki, 54124, Thessaloniki; [2]Natural Products Research Centre of Excellence-AUTH (NatPro-AUTH), Center for Interdisciplinary Research and Innovation (CIRI-AUTH), 57001 Thessaloniki, Greece;

*Corresponding author.

E-mail addresses: ioannapyrka@chem.auth.gr (Ioanna Pyrka), niknen@chem.auth.gr (Nikolaos Nenadis).

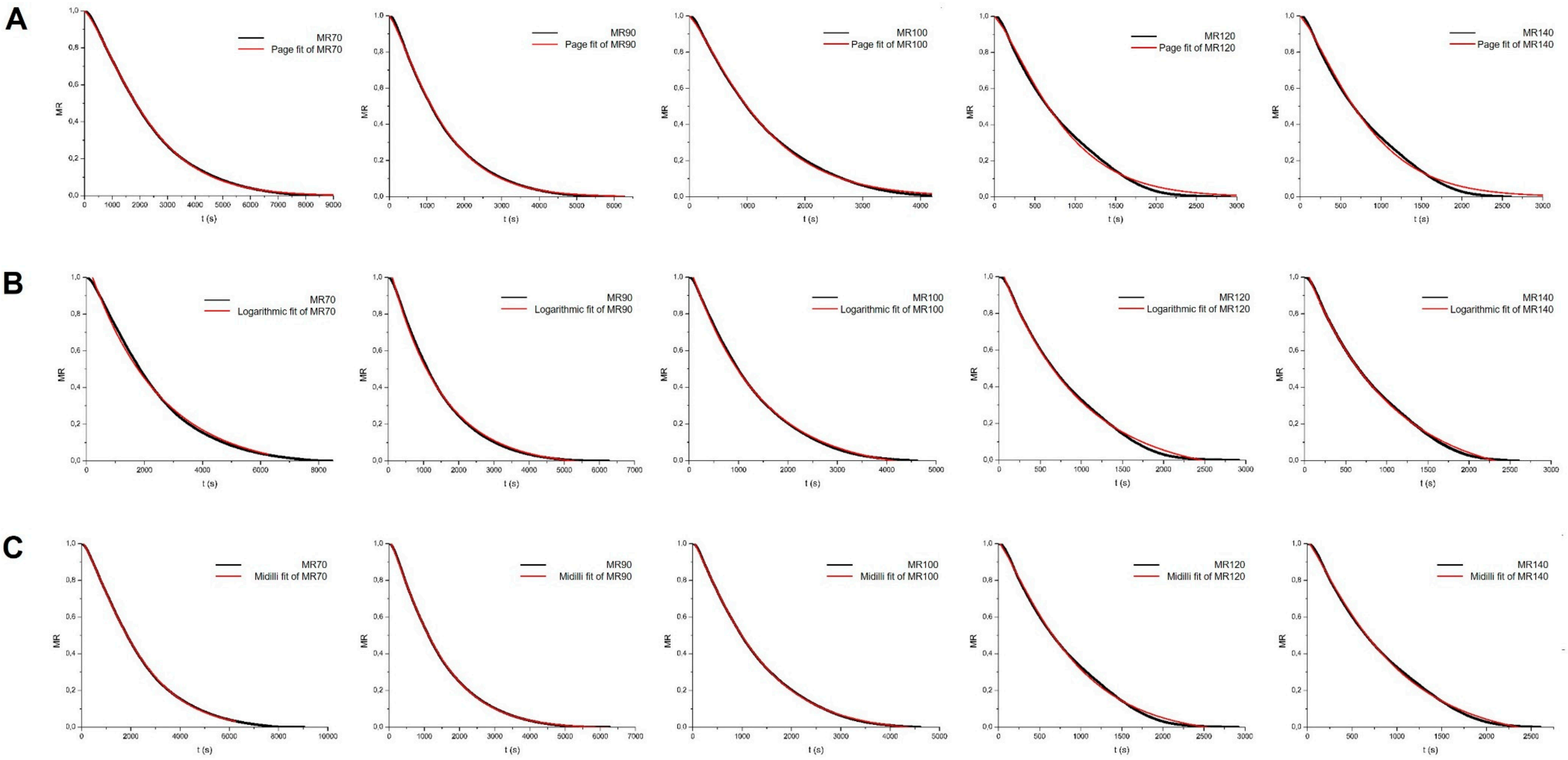

Figure S1. Page (A), logarithmic (B) and Midilli (C) kinetic models adjusted to experimental drying curves at 70, 90, 100, 120 and 140 °C.

Table S1
Effective diffusivity at the studied temperatures.

Temperature	70 °C	90 °C	100 °C	120 °C	140 °C
D_{eff} (m^2/s)	1.417×10^{-9}	2.231×10^{-9}	2.637×10^{-9}	4.361×10^{-9}	5.807×10^{-9}
